# Nurses’ perspectives on working conditions while caring for patients with COVID-19 during the epidemic threat in Poland

**DOI:** 10.3389/fpubh.2025.1581671

**Published:** 2025-07-07

**Authors:** Lidia Elżbieta Sierpińska, Monika Mikołajewska, Elżbieta Araminowicz-Kierklo, Łukasz Rydzik, Magdalena Makarska-Białokoz, Wojciech Czarny

**Affiliations:** ^1^Military Clinical Hospital No. 1 with Polyclinic, Independent Public Health Care Unit, Lublin, Poland; ^2^Faculty of Health Sciences, Radom Higher School, Radom, Poland; ^3^Faculty of Health Sciences, Vincent Pol University, Lublin, Poland; ^4^Faculty of Health Sciences, Students’ Scientific Circle, Radom Higher School, Radom, Poland; ^5^Faculty of Health Sciences, Uinversity of Warmia and Mazury, Olsztyn, Poland; ^6^Faculty of Physical Education and Sport, Institute of Sports Sciences, University of Physical Culture, Krakow, Poland; ^7^Department of Beauty Sciences, Faculty of Health Sciences, Vincent Pol University, Lublin, Poland; ^8^College of Medical Sciences, Institute of Physical Culture Studies, University of Rzeszow, Rzeszow, Poland

**Keywords:** nursing workforce, occupational stress, personal protective equipment, staffing shortages, hospital management, COVID-19

## Abstract

**Introduction:**

The severe state of health of patients with coronavirus disease 2019 (COVID-19) has increased the risk of arduous and hazardous work conditions for nurses. The aim of this study is to identify the conditions of nurses’ work during the care of patients with COVID-19 during the state of epidemic threat.

**Methods:**

The COVID-19 pandemic has significantly impacted the healthcare system, especially that of frontline nurses. This study aimed to assess the work conditions and psychological stress experienced by nurses during the pandemic. This study included 116 nurses who provided care to patients with COVID-19 in hospital wards. A random sampling method was employed to select participants from among those working in the shift system. A diagnostic survey method was used to collect data, with a focus on work conditions, stress exposure, and the provision of personal protective equipment (PPE). Data analysis was performed using SPSS v.29 (Statistical Package for the Social Sciences, Version 29), and chi-squared and Fisher’s exact tests were applied.

**Results:**

Of the total nurses, 69.0% reported that they were ‘sometimes’ provided with personal protective equipment. According to 60.3% of participants, nurse staffing was provided in accordance with regulations ‘sometimes.’ A total of 64.7% of the participants experienced stress: concern about infection with SARS-CoV-2, severe health status of patients, patient death, and shortage of equipment. The main complaints related to stress were difficulty concentrating, sleep problems, and headaches; 69.8% of the participants were exposed to hazardous factors at work, and 69.0% experienced arduous factors. Shortages of medical equipment were reported, mainly because of the lack of respirators, cardiac monitors, and inhalators.

**Conclusion:**

Nurses employed in hospital wards during the care of patients with COVID-19 worked under difficult conditions. Sometimes, they were provided with personal protective equipment, and sometimes, nurse staffing was provided in accordance with regulations. There was significant exposure to stress, hazardous and arduous factors, and shortages of medical equipment.

## 1 Introduction

Coronavirus disease 2019 (COVID-19) is caused by infection with SARS-CoV-2 (severe acute respiratory syndrome coronavirus 2), a betacoronavirus of zoonotic origin. It is presumed that Asian Rhinolophus bats, consumed in some regions of China, were responsible for the initial transmission of the virus to humans, with the seafood market in Wuhan identified as the likely site of the zoonotic spillover. The first confirmed case of SARS-CoV-2 infection was reported on December 1, 2019. The virus spread rapidly; by the end of December 2019, over 80,000 cases were registered in China (1). In Poland, the first case of COVID-19 was reported on March 4, 2020 (2). As of July 2022, more than 122 million infections had been confirmed worldwide, including 4.2 million in Europe and over 2 million in Poland. The global death toll exceeded 2.7 million, with 49,300 deaths reported in Poland (3).

COVID-19 has an incubation period of up to 2 weeks, during which asymptomatic individuals may unknowingly transmit the virus (4). The infection typically begins in the upper respiratory tract (nose, throat, and larynx) and may progress to the lower respiratory tract, affecting the pulmonary alveoli. SARS-CoV-2 has also been detected in other organs, including the central nervous system, heart, pancreas, kidneys, gastrointestinal tract, blood vessels, and male reproductive organs, resulting in a diverse range of clinical symptoms. The absence of fully established therapeutic protocols and the impact of comorbidities further complicate treatment (5). Disease severity varies depending on the stage of illness, patient age, and general health, with older adults and those with chronic conditions being particularly vulnerable (6).

One of the most serious consequences of COVID-19 is hypoxia—a reduction in blood oxygen saturation. In such cases, hospitalization is necessary to administer oxygen therapy. Patients with severe forms of the disease may develop acute respiratory failure requiring high-flow oxygen therapy or mechanical ventilation (7).

Nurses have played a central role in the hospital care of patients with COVID-19. Studies indicate that nurses were the healthcare professionals most frequently in direct contact with infected individuals (8). In Poland, one study confirmed that shift work during the pandemic contributed significantly to stress levels among nurses. Staff shortages often led to increased workloads and frequent overtime (9). Similarly, international research has shown that the pandemic negatively affected the physical and mental health of healthcare workers (10).

Key factors that mitigate the burden on nurses include the reliable provision of personal protective equipment (PPE), such as gowns, masks, gloves, goggles, and footwear, which meet established safety standards (11). Equally important is the availability of medical equipment. During the pandemic, shortages of respirators, oxygen masks, and inhalation devices were commonly reported (12).

The pandemic affected nurses on multiple levels—physically (increased workload and fatigue), mentally (stress and anxiety), and socially (isolation from family and support networks), as widely documented in international literature. This study is grounded in prior research on occupational stress, burnout, and safety in healthcare settings during pandemics, including evidence from previous coronavirus outbreaks. In Poland, public health measures included mandatory COVID-19 vaccination for healthcare workers, regular surveillance testing (e.g., PCR or rapid antigen swabs), and isolation protocols in the case of infection. These national policies have significantly influenced infection control practices and risk perception in clinical environments. Therefore, this study aimed to assess the working conditions experienced by nurses providing care to patients with COVID-19 in hospital wards during the period of the declared epidemic threat in Poland. The main research questions were (1) What work conditions did nurses experience while caring for patients with COVID-19? (2) Which occupational stressors and resource shortages were reported most frequently? It seeks to assess the working conditions experienced by nurses providing care to patients with COVID-19 in Poland during the period of the declared epidemic threat. The data for this study were collected from February to March 2023, a period corresponding to the late phase of the COVID-19 pandemic in Poland. This time frame aligns with what has been referred to in international literature as the “post-Omicron” phase—characterized by high vaccination coverage (including booster doses), ongoing but less severe community transmission, and reduced hospitalization and mortality rates. Although national-level public health policies, such as mandatory healthcare worker vaccinations and isolation protocols, remained in effect, the epidemiological context differed markedly from the earlier pandemic phases.

## 2 Materials and methods

### 2.1 Population and research project

This study included 116 nurses who, during the state of epidemic threat due to COVID-19, provided care to patients in hospital wards at Mazovian Specialist Hospital in Radom, Poland. Due to infection with SARS-CoV-2, patients were isolated in various hospital wards (conservative and surgical). The study process was preceded by obtaining consent from the manager of the institution. The ages of the nurses in the study ranged from 24 to 59 years. In the examined group, females predominated (94.8%) compared to males (5.2%). Marital status and family responsibilities (e.g., having children or economic dependents) were not collected, which could be considered a limitation of this study. This study was conducted from February 1 to March 3, 2023. The research project was submitted to the Dean’s Office at the Radom Higher School in Radom (Radomska Szkoła Wyższa [RSW]) by the co-author of the research project—a member of the Students’ Scientific Circle at the RSW in Radom (Catalog No. 12857/2022), and consent for the study was obtained from the Dean of the RSW in Radom.

### 2.2 Selection of the study group

The nurses were randomly selected for the study. The criterion for sample selection was nurses’ work in hospital wards (conservative and surgical), where 24-h care was provided to adults diagnosed with COVID-19. Into the study were qualified exclusively the nurses who took round-the-clock care of patients in a shift system, during the day and at night. The exclusion criterion was nursing management staff who did not provide direct care to patients with COVID-19 and did not work on shift duties. Apart from this, the study excluded nurses employed in a single-shift system in hospital wards, those working in surgical rooms/diagnostic laboratories, or those employed in management positions who were not engaged in direct, complex care of patients isolated due to infection with SARS-CoV-2. Random sampling was used to select participants from an available pool of nurses working in hospital wards during the pandemic. Nurses were randomly chosen based on their shift schedules. Efforts were made to minimize bias by ensuring random selection of participants and conducting the study anonymously to avoid any pressure on the participants.

### 2.3 Method and research instrument

This study was conducted using a diagnostic survey and an author-constructed questionnaire designed for the purpose of this study. The questionnaire was author-constructed due to the lack of a standardized, validated instrument for this study. However, the development process involved expert input, and a pilot test was conducted to ensure the clarity and relevance of the questions. The questionnaire consisted of closed questions systematized according to seven domains, which are summarized in the Table 1.

**Table 1:** Structure of the author-constructed questionnaire with domains and corresponding questions concerning nurses’ working conditions during the COVID-19 epidemic threat.

Domain	Questions
Domain I: Provision of personal protective equipment during the care of patients with COVID-19	1. Was personal protective equipment provided in the ward? 2. If ‘sometimes,’ which items of personal protective equipment were missing? 3. Was the training conducted in the ward regarding the use of personal protective equipment?
Domain II: Adjustment of nurse staffing to the demand for care during the care of patients with COVID-19	1. Was nurse staffing provided in the ward in accordance with the regulations? 2. If ‘sometimes,’ on what duties did shortages occur? 3. Did you work overtime?
Domain III: Exposure to stress in the work of nursing staff during the care of patients with COVID-19	1. Did you experience stress during the care of patients with COVID-19? 2. If stress ‘occurred,’ what were its causes? 3. In case of stress, what complaints did you experience?
Domain IV: Exposure to arduous factors in the work of nursing staff during the care of patients with COVID-19	1. In your opinion, did arduous factors occur during the care of patients with COVID-19? 2. If such factors ‘occurred,’ what were they?
Domain V: Exposure to hazardous factors in the work of nursing staff during the care of patients with COVID-19	1. In your opinion, did hazardous factors occur during the care of patients with COVID-19? 2. If such factors ‘occurred,’ what were they?
Domain VI: Provision of wards with medical equipment/devices during the care of patients with COVID-19	1. Was the ward provided with medical equipment/devices during the care of patients with COVID-19? 2. If ‘sometimes,’ what did the shortages concern?
Domain VII: Demographic and social data	1. What is your gender? 2. What is your age? 3. What is your level of education?

The theoretical foundation for developing the questionnaire was based on existing studies on occupational stress, burnout, and personal protective equipment in healthcare settings during pandemics. These studies guided the construction of the domains and questions in the survey to ensure that the instrument addressed the relevant issues faced by healthcare workers during the COVID-19 pandemic. This study was preceded by a pilot study to verify the author-constructed questionnaire. After testing the research instrument, it was assessed that the questions and instructions for the participants were understandable and that the instrument was correctly constructed. The survey instrument was pilot-tested with a small group of nurses who were not included in the main study. Feedback from the pilot study was used to refine the questions and improve their clarity. The development process also involved expert input to ensure that the instrument was suitable for the study. The theoretical foundation for developing the questionnaire was based on prior studies concerning occupational health risks, stress, and protective measures among healthcare workers during the pandemic. The questionnaire did not include items related to prior SARS-CoV-2 infection or vaccination status, or these variables were not considered in the analysis and should be acknowledged as limitations of the study.

#### 2.3.1 Survey administration details

A questionnaire was administered in person to each nurse during scheduled work breaks in the hospital ward. Participation was voluntary and anonymous. A member of the research team provided standardized instructions on how to complete the form and remained available to clarify any doubts. The respondents completed the questionnaire independently, without time pressure, and returned it in sealed envelopes to ensure confidentiality. On average, the completion took approximately 15–20 min. No identifying information was collected, and the data were entered into the database by two researchers to minimize transcription errors.

### 2.4 Statistical analysis

The collected data were analyzed using the chi-squared test, or in the case of 2×2 tables, Fisher’s exact test for small-sized groups. Statistical analyses were performed using SPSS v. 29 (Statistical Package for the Social Sciences, Version 29). The *p*-values of <0.05 were considered statistically significant.

## 3 Results

### 3.1 Characteristics of the study group

This study included 116 nurses. Tables 2, 3 present participants’ sociodemographic characteristics. An age of 50 years was used as a reference point because it represented the median age of the sample and allowed for a more meaningful subgroup analysis.

**Table 2:** Structure of the nurses examined according to gender, education, and age.

Variable	Category	Age						*p*
		<50		50 and over		Total		
		*N*	%	*N*	%	*N*	%	
Gender	Females	49	89.1	61	100.0	110	94.8	0.01^a^
	Males	6	10.9	–	–	6	5.2	
Education	Secondary school	–	–	9	14.8	9	7.8	0.006^b^
	Higher—Licentiate	32	58.2	32	52.5	64	55.2	1.000^c^
	Higher—Master’s degree	23	41.8	20	32.8	43	37.1	

a Fisher’s exact test.

b Fisher’s exact test in combination with secondary school education and higher education (licentiate + master’s degree). The results were adjusted using the Bonferroni correction for two comparisons.

c Fisher’s exact test in combination with higher licentiate education versus master’s degree (omitting secondary school education). The result was adjusted by the Bonferroni correction for two comparisons.

**Table 3:** Structure of the nurses examined according to age.

Variable	*N*	*M*	SD	Me	Min.	Max.
Age (years)	116	48.7	9.74	50.0	25	67

*N*, number of observations; *M*, mean; SD, standard deviation; Me, median; Min., minimum value; Max., maximum value.

### 3.2 Nurses’ perspectives on work conditions in the care of patients with COVID-19

Table 4 presents the results of the analysis of nurses’ responses regarding their working conditions while caring for patients with COVID-19.

**Table 4:** Participants’ opinions concerning work conditions in the care of patients with COVID-19 in hospital wards according to age.

Variable	Categories	Age						*p*
		<50		50 and over		Total		
		*N*	%	*N*	%	*N*	%	
I.1. Was personal protective equipment provided in the ward?	No	2	3.6	–	–	2	1.7	< 0.001^A^
	Sometimes	27	49.1	53	86.9	80	69.0	
	Always	26	47.3	8	13.1	34	29.3	
	Total	55	100.0	61	100.0	116	100.0	
I.3. Was the training conducted in the ward regarding the use of personal protective equipment?	No	23	41.8	10	16.4	33	28.4	< 0.001
	Sometimes	14	25.5	43	70.5	57	49.1	
	Yes	18	32.7	8	13.1	26	22.4	
	Total	55	100.0	61	100.0	116	100.0	
II.1. Was nurse staffing provided in the ward in accordance with regulations?	Not adjusted	18	32.7	16	26.2	34	29.3	0.201
	Adjusted sometimes	29	52.7	41	67.2	70	60.3	
	Adjusted (according to regulations—0.7 or 0.6 full-time job per 1 bed)	8	14.5	4	6.6	12	10.3	
	Total	55	100.0	61	100.0	116	100.0	
II.3. Did you work overtime?	No	12	21.8	3	4.9	15	12.9	0.007
	Sometimes	11	20.0	24	39.3	35	30.2	
	Yes	32	58.2	34	55.7	66	56.9	
	Total	55	100.0	61	100.0	116	100.0	
III.1. Did you experience stress during the care of patients with COVID-19?	Did not occur	1	1.8	1	1.6	2	1.7	0.003^A^
	Occurred sometimes	11	20.0	28	45.9	39	33.6	
	Occurred	43	78.2	32	52.5	75	64.7	
	Total	55	100.0	61	100.0	116	100.0	
IV.1. In your opinion, did arduous factors occur during the care of patients with COVID-19?	Occurred sometimes	8	14.5	28	45.9	36	31.0	< 0.001
	Occurred	47	85.5	33	54.1	80	69.0	
	Total	55	100.0	61	100.0	116	100.0	
V.1. In your opinion, did hazardous factors occur during the care of patients with COVID-19?	Did not occur	–	–	1	1.6	1	0.9	< 0.001^A^
	Occurred sometimes	8	14.5	26	42.6	34	29.3	
	Occurred	47	85.5	34	55.7	81	69.8	
	Total	55	100.0	61	100.0	116	100.0	
VI.1. Was the ward provided with equipment/devices during the care of patients with COVID-19?	No	7	12.7	5	8.2	12	10.3	0.116
	Sometimes	36	65.5	50	82.0	86	74.1	
	Always	12	21.8	6	9.8	18	15.5	
	Total	55	100.0	61	100.0	116	100.0	

A Analyses using χ^2^ tests were performed, omitting the small category ‘No’.

### 3.3 Assessment of the provision of nurses in the ward with personal protective equipment

Each nurse in the study was asked whether personal protective equipment was provided in the ward. It was found that the majority of nurses providing care to patients with COVID-19 reported that they were provided personal protective equipment ‘sometimes’ (*n* = 80; 69.0%), followed by ‘always’ (*n* = 34; 29.3%), whereas the remaining participants mentioned ‘No’ (*n* = 2; 1.7%).

**Figure 1:**
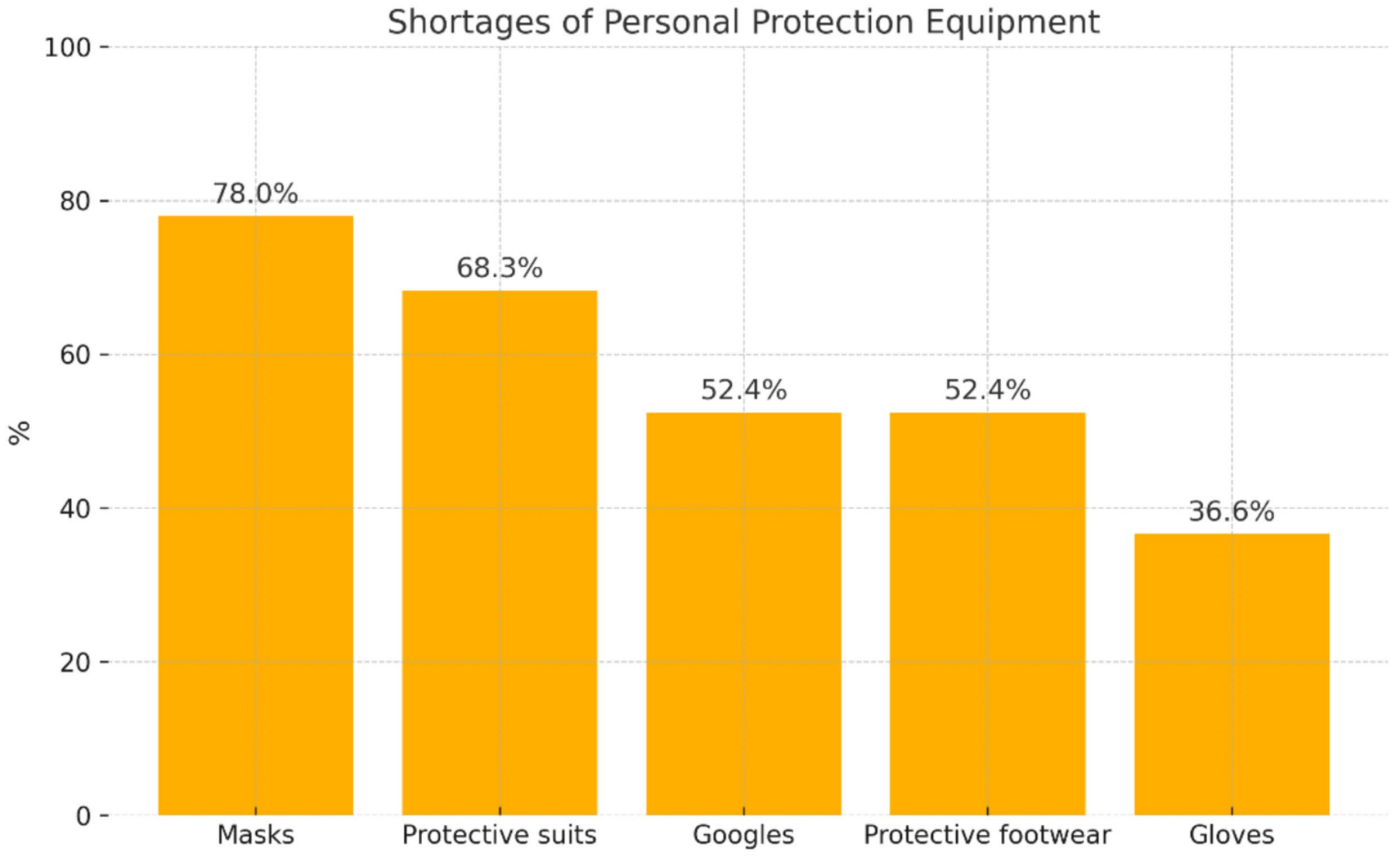
Distribution of the assessment of shortages ‘sometimes’ of personal protective equipment.

Nurses who admitted that personal protective equipment was provided ‘sometimes’ or not provided (*n* = 82) reported what the shortages concerned (Figure 1).

It was found that the largest number of nurses from this group reported shortages of ‘masks’ (*n* = 64; 78%) and ‘protective suits’ (*n* = 56; 68.3%). More than half of the participants from this group reported the shortage of ‘goggles’ (*n* = 43; 52.4%) and ‘protective footwear’ (*n* = 43; 52.4%), while ^1^/_3_ of nurses in this group mentioned a shortage of ‘gloves’ (*n* = 30; 36.6%).

It was considered important to recognize whether trainings were carried out in the ward regarding the use of personal protective equipment. Nearly half of the examined nurses reported that trainings in the use of personal protective equipment were carried out ‘sometimes’ in the ward (*n* = 57; 49.1%), 28.4% of participants mentioned the lack of such trainings (*n* = 33), while the remainder confirmed their organization (*n* = 26; 22.4%).

### 3.4 Assessment of the provision of nurses in accordance with regulations in effect

The opinions of nurses regarding work conditions in the care of patients with COVID-19 were also analyzed from the perspective of providing proper nurse staffing in accordance with the regulations in effect. In the opinions of the majority of participants, nurse staffing was ‘sometimes’ adjusted to the regulations in effect (*n* = 70; 60.3%). The lack of adjustment of the staffing was indicated by 29.3% of participants (*n* = 34), whereas a small group of nurses in the study confirmed that nurse staffing in the wards where patients with COVID-19 were treated was in accordance with regulations, i.e., 0.7 or 0.6 full-time/1 bed (*n* = 12; 10.3%).

**Figure 2:**
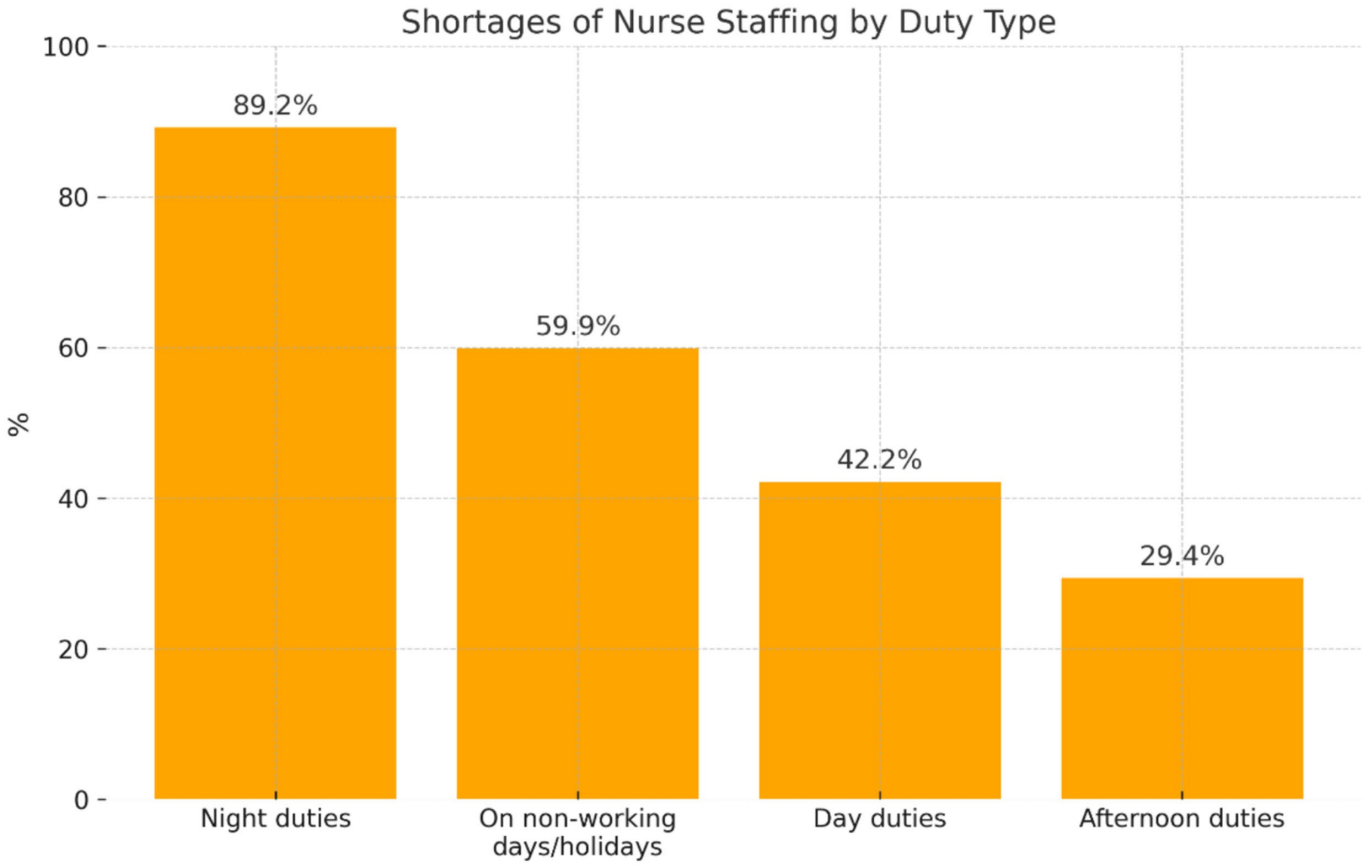
Distribution of the participants’ answers concerning shortages of nurse staffing on various duties, at different times of the day, and on non-working days/holidays.

The analysis of the research material included answers provided by 102 nurses in the study to the question of what duties there were shortages of nurse staffing (Figure 2).

Participants who reported that proper nurse staffing was provided ‘sometimes’ most frequently indicated the occurrence of shortages on night duties (*n* = 91; 89.2%). As a justification, the nurses mentioned that some of them could not work at night due to health reasons, pregnancy, or care of a child under 4. Shortages also occurred on ‘non-working days/holidays’ (*n* = 61; 59.8%)—some nurses had to take care of a child, and on day duties (*n* = 43; 42.2%). Afternoon shifts were rarely reported (*n* = 30; 29.4%). According to the participants, the deficit in nurse staffing on various duties was also due to sick absenteeism among nurses infected with SARS-CoV-2, pain in the musculoskeletal system due to physical overload, high-risk pregnancy, and staff fluctuation.

In this study, it was also determined whether nurses worked overtime. In the majority of cases, the participants worked overtime (*n* = 66; 56.9%); ^1^/_3_ of the study participants (*n* = 35; 30.2%) worked overtime ‘sometimes,’ whereas a part of them denied that they worked overtime (*n* = 15; 12.9%).

### 3.5 Assessment of exposure of nurses to stress during the care of patients with COVID-19

The collected research material was analyzed from the perspective of the occurrence of stress in nurses during the care of patients with COVID-19. The majority of participants confirmed that they experienced stress during the care of patients with COVID-19 (*n* = 75; 64.7%); 33.6% of them mentioned that stress occurred ‘sometimes’ (*n* = 39; 33.6%), while 1.7% of participants admitted that stress ‘did not occur’ (*n* = 2).

**Figure 3:**
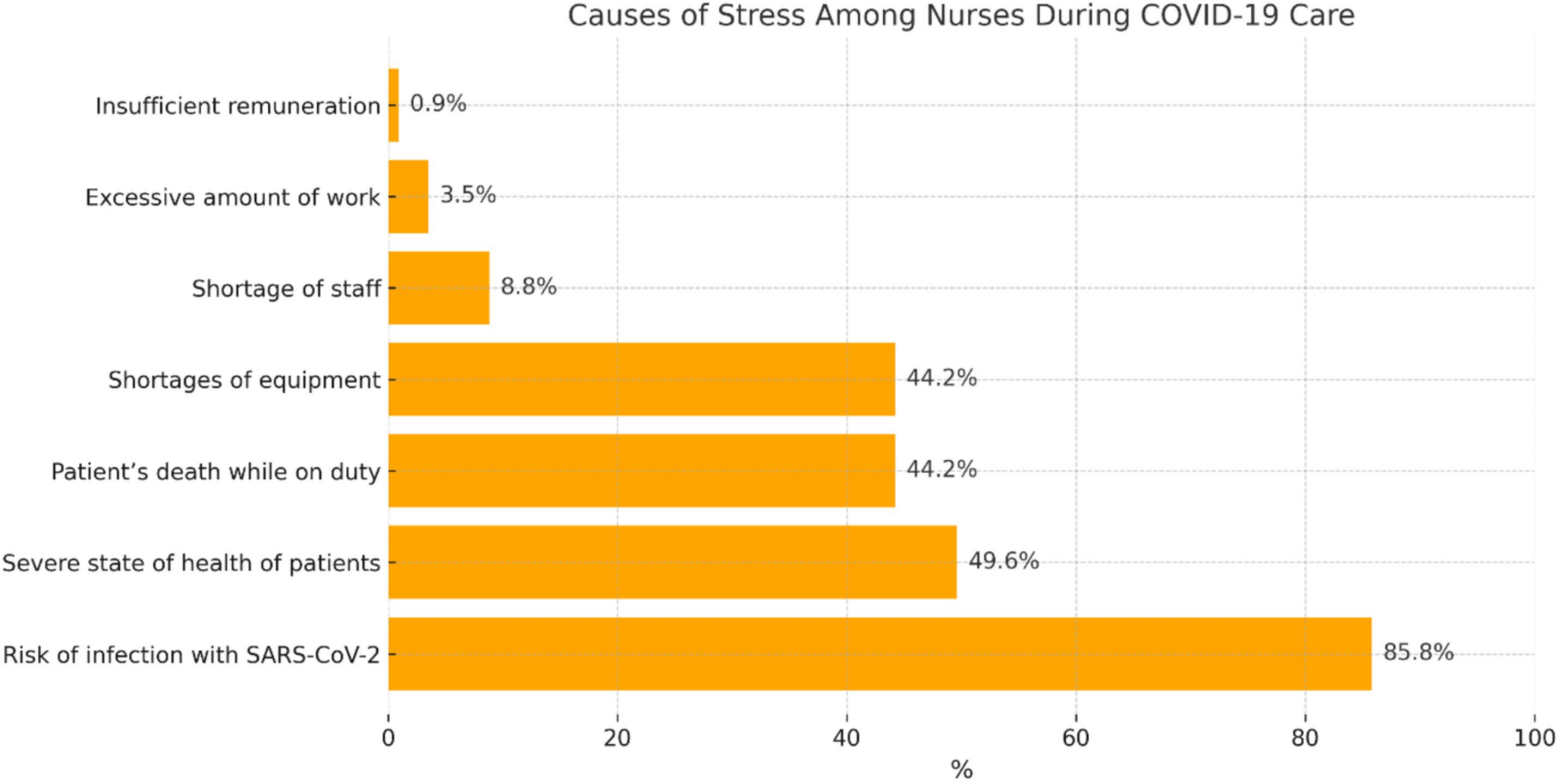
Distribution of the participants’ answers concerning the causes of stress.

Participants who experienced stress were asked about the causes of its occurrence during the care of patients with COVID-19. A total of 113 participants provided their answers (Figure 3).

Participants who confirmed the occurrence of stress during the care of patients with COVID-19 most often mentioned that it was related to the ‘risk of infection with SARS-CoV-2’ (*n* = 97; 85.8%). They reported that concerns about infection also concerned their families and other significant people. A considerable percentage of participants indicated such stressors as ‘severe state of health of patients’ (*n* = 56; 49.6%), ‘shortage of equipment’ (*n* = 50; 44.2%), and ‘patient’s death while on duty’ (*n* = 50; 44.2%), whereas they more rarely indicated ‘shortage of staff’ (*n* = 10; 8.8%), ‘excessive amount of work’ (*n* = 4; 3.5%), and ‘insufficient remuneration’ (*n* = 1; 0.9%).

**Figure 4:**
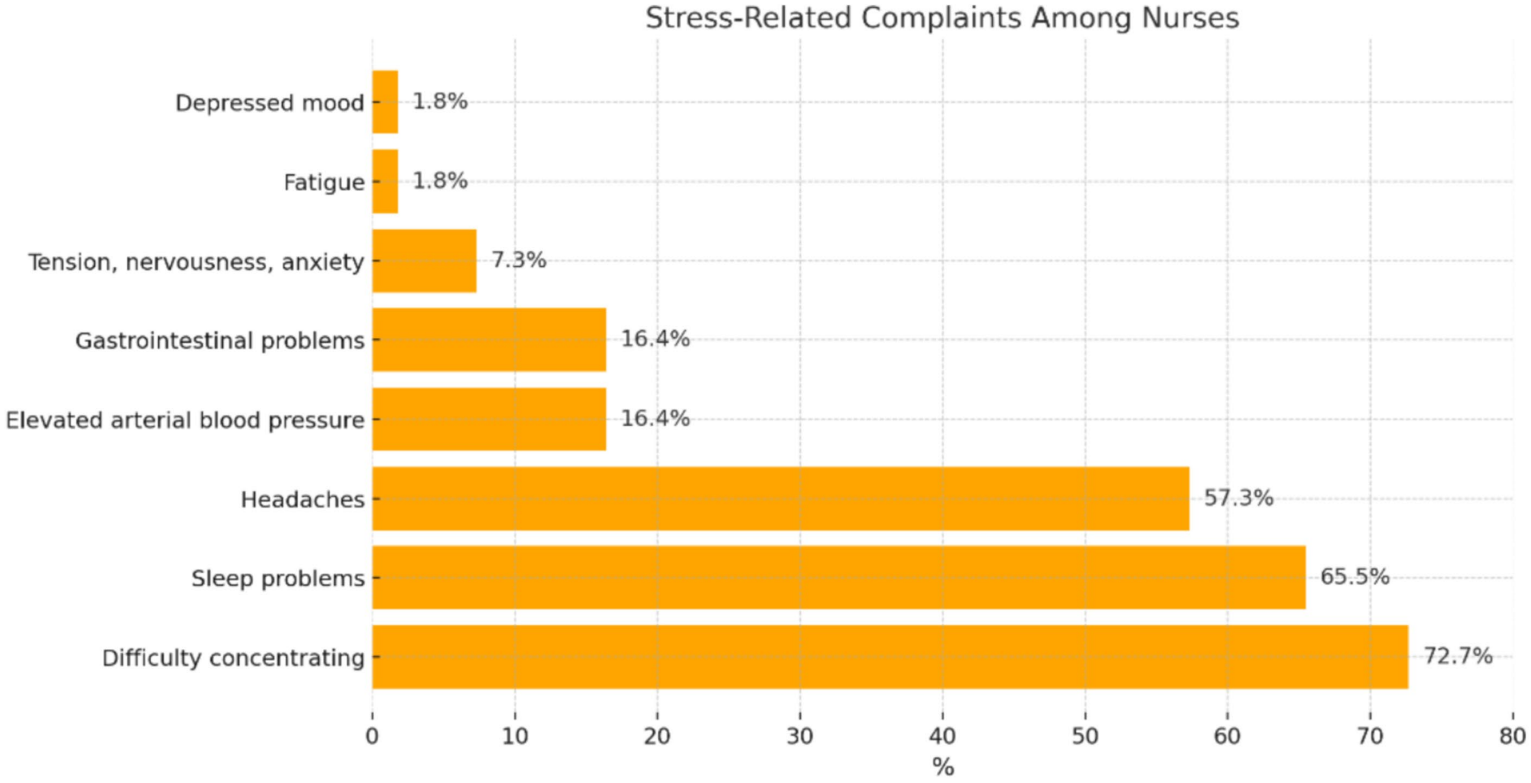
Distribution of participants’ answers concerning complaints experienced in relation to the occurrence of stress.

The nurses were asked about complaints experienced in relation to the occurrence of stress (Figure 4).

Participants who experienced stress in relation to the care of patients with COVID-19 most often experienced complaints, such as: ‘difficulty concentrating’ (*n* = 80; 72.7%), ‘sleep problems’ (*n* = 72; 65.5%), and ‘headaches’ (*n* = 63; 57.3%). Rarely reported complaints were ‘elevated arterial blood pressure’ (*n* = 18; 16.4%), ‘gastrointestinal problems’ (*n* = 18; 16.4%), ‘difficulty concentrating’ (*n* = 2; 1.8%), and ‘tension, nervousness, and anxiety’ (*n* = 8; 7.3%). The occurrence of ‘fatigue’ or ‘depressed mood’ was reported by 1.8% of the participants each.

### 3.6 Assessment of exposure of nurses to hazardous factors during the care of patients with COVID-19

Each nurse in the study was asked about their exposure to hazardous factors during the care of patients with COVID-19. The majority of participants confirmed the ‘occurrence’ of hazardous factors while providing care to patients with COVID-19 (*n* = 81; 69.8%). The remaining participants indicated the answers ‘occurred sometimes’ (*n* = 34; 29.3%) or ‘did not occur’ (*n* = 1; 0.9%).

**Figure 5:**
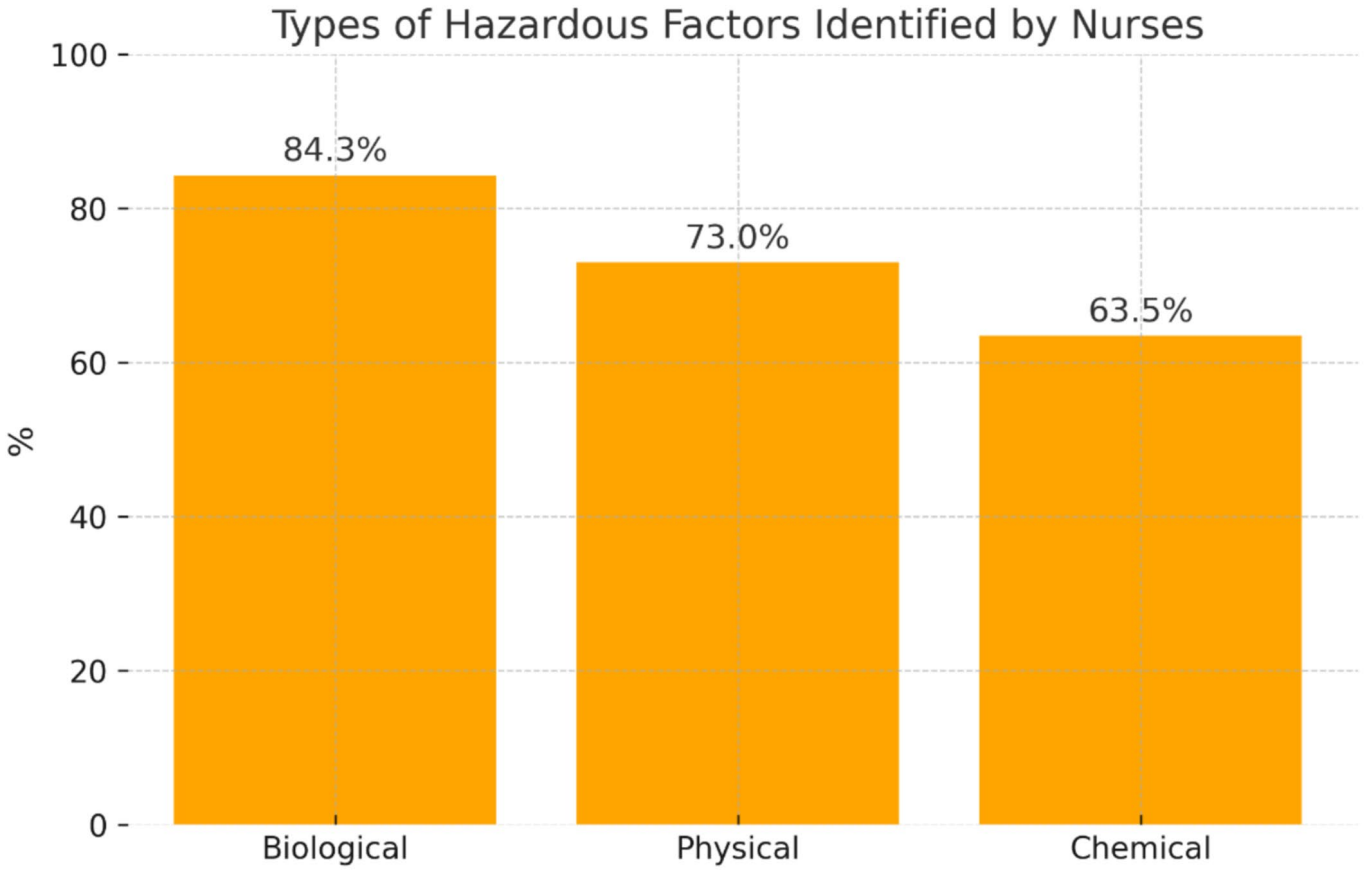
Distribution of participants’ answers regarding the types of hazardous factors.

It was found that 115 participants indicated the hazardous factors that occurred in the work of nurses during the care of patients with COVID-19 (Figure 5).

The participants frequently mentioned the occurrence of hazardous ‘biological’ factors (*n* = 97; 84.3%). The nurses reported mainly the risk of infection with SARS-CoV-2, bacteria, and fungi, while they slightly less frequently indicated ‘physical’ factors (*n* = 84; 73.0%)—failure of electrical equipment, slippery surfaces, and ‘chemical’ factors—disinfectants (*n* = 73; 63.5%).

### 3.7 Exposure of nurses to arduous factors during the care of patients with COVID-19

The study also included the recognition that nurses caring for patients with COVID-19 in hospital wards were exposed to arduous factors. The analysis of data showed that the largest number of the examined nurses confirmed the occurrence of arduous factors during the care of patients with COVID-19 (*n* = 80; 69.0%), while the remainder considered that these factors occurred ‘sometimes’ (*n* = 36; 31.0%).

**Figure 6:**
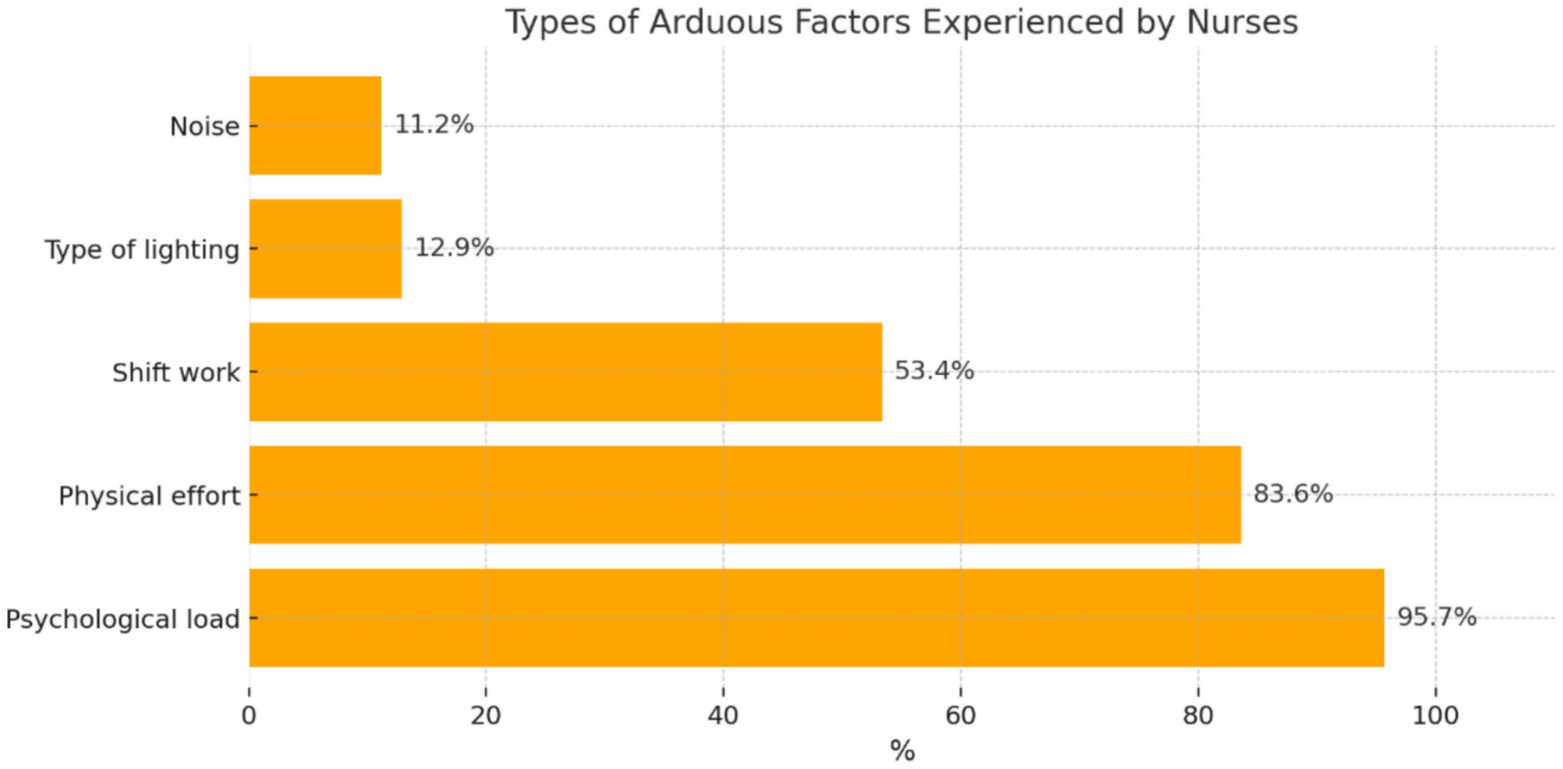
Distribution of participants’ answers regarding the types of arduous factors.

The participants were asked about the type of arduous factors most often occurring in the care of patients with COVID-19 (Figure 6).

The participants who confirmed the occurrence of arduous factors during the care of patients with COVID-19 most frequently reported ‘psychological load’ (*n* = 111; 95.7%), followed by ‘physical effort’ (*n* = 97; 83.6%), ‘shift work’ (*n* = 62; 53.4%), ‘type of lighting’ (*n* = 15; 12.9%), and ‘noise’ (*n* = 13; 11.2%).

### 3.8 Provision of medical equipment/devices in the wards where patients with COVID-19 were treated

The author-constructed questionnaire contained questions concerning the recognition of whether the ward where patients with COVID-19 were treated was properly provided with medical equipment/devices. The majority of participants admitted that during the care of patients with COVID-19, the ward was provided with proper equipment/devices ‘sometimes’ (*n* = 86; 74.1%); 15.5% of the examined nurses mentioned that the ward was ‘always’ properly equipped (*n* = 18), whereas the remaining participants indicated the answer ‘No’ (*n* = 12; 10.3%).

**Figure 7:**
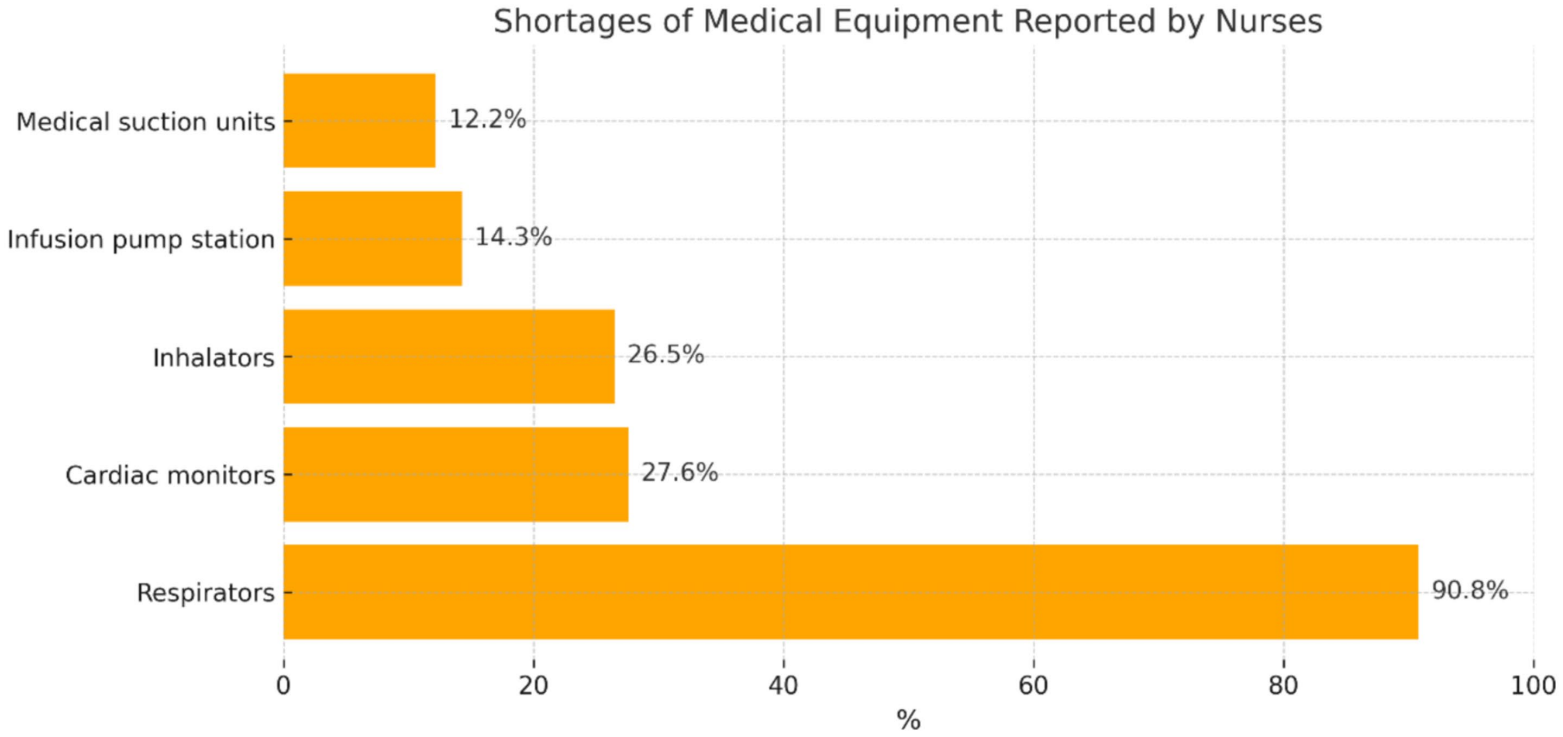
Distribution of participants’ answers regarding shortages of medical equipment/devices.

The participants were asked to indicate shortages of medical equipment/devices in the wards where patients with COVID-19 were treated (Figure 7).

The participants who reported that sometimes there were shortages of medical equipment during the care of patients with COVID-19 most frequently mentioned the lack of ‘respirators’ (*n* = 89; 90.8%), and more rarely shortages of ‘cardiac monitors’ (*n* = 27; 27.6%), ‘inhalators’ (*n* = 26; 26.5%), ‘infusion pump stations’ (*n* = 14; 14.3%), and ‘medical suction units’ (*n* = 12; 12.2%).

## 4 Discussion

The COVID-19 pandemic was a difficult situation for the medical environment due to a lack of knowledge about the new disease, the speed of transmission of the disease by airborne droplets, non-specific symptoms, SARS-CoV-2 mutations, and a severe course of infection in patients with concomitant diseases (12, 13).

During the COVID-19 pandemic, healthcare workers were exposed to difficult work conditions. Physical load was accompanied by the risk of infection with SARS-CoV-2, increased level of stress, feeling of helplessness, necessity for limiting contacts with close ones, and fear about one’s own health and the health of the family (14). Our quantitative findings concerning stress, PPE shortages, and insufficient staffing are consistent with qualitative research by Maghsoodi et al. (15), who described nurses’ experiences during the COVID-19 pandemic as shaped by an “unsafe work environment” and the “shadow of suffering and death.” Their study emphasized emotional exhaustion, perceived managerial neglect, and long-term mental strain, enriching the understanding of the issues reported in our survey data, particularly stress symptoms and psychological burdens. The combination of both perspectives allows for a more comprehensive view of nurses’ experience during an epidemic. Therefore, it is very important to provide proper work conditions in healthcare in accordance with the standards, with particular attention to the work of nurses (16–18).

The above-mentioned aspects provided incentives for research in order to recognize the conditions of the work of nurses in the care of patients with COVID-19 in hospital conditions.

The study group consisted of 116 nurses employed in the Mazovian Specialist Hospital in Radom, Poland, during the period of epidemic threat due to COVID-19.

A study conducted by Ginter et al. demonstrated a correlation between the availability of personal protective equipment and the level of anxiety among nursing staff. The better the availability of masks, gloves, goggles, and protective suits, the lower the occurrence of anxiety disorders (19). The present study showed that the participants most often reported that personal protective equipment was provided in the ward ‘sometimes’ (69%). According to 29.3% of the study participants, this equipment was provided ‘always.’ The remaining participants admitted that personal protective equipment was not provided in the wards where they worked (1.7%). The participants who mentioned that personal protective equipment was provided ‘sometimes’ most often indicated shortages of masks (78.0%) and protective suits (68.3%), while they more rarely reported shortages of goggles (5.4%), protective footwear (52.4%), and gloves (36.6%).

Based on the relevant literature, persons who presented a higher level of knowledge concerning the ways of transmission of the virus and prevention of infection with SARS-CoV-2 were less susceptible to occupational burnout and the level of anxiety (20). According to the official statement by the Polish Nurses Association, attention was paid to the necessity of developing new standards of management and training medical staff in the event of a pandemic (21). The present study showed that approximately half of the study participants reported that training in the use of personal protective equipment in the ward was ‘sometimes’ carried out (49.1%). A lack of such training was mentioned by 28.4% of the participants, whereas the remainder confirmed their organization (22.4%).

In the opinion of Ciesielska, overload while performing professional activities results in a decline in satisfaction with work and quicker occupational burnout (22)^.^ Iranian researchers confirmed that the adjustment of nurse staffing to the demand for care during the care of patients with COVID-19 is important for, among other things, the prevention of occupational burnout and further professional activity (23). According to legal regulations, nurse staffing should be adjusted to meet the demands for nursing care. Our study demonstrated that nurse staffing was ‘sometimes’ adjusted to the regulations in effect (60.3%); 29.3% of participants admitted a lack of such an adjustment, while 10.3% of the nurses in the study mentioned that the staffing was in accordance with regulations, i.e., 0.7 or 0.6 full-time jobs/1 bed (10.3%). Nurses who reported that the proper nurse staffing was provided ‘sometimes’ most often indicated shortages in night duties (86.7%), non-working days/holidays (58.1%), and morning duties (41%). Afternoon duties were rarely reported (28.6%). The majority of participants worked overtime (56.9%).

According to Leppert et al., stress at work is the factor that most strongly generates occupational burnout and decreases satisfaction with work (24). According to Tomaszewska and Lasota, nurses are exposed to stress during the COVID-19 pandemic (25). The researchers (Wang, Farokhnia, and Sanchuli) found that the COVID-19 pandemic exerted a negative effect on the psychological health of society, including medical staff (26). A team of Iranian researchers confirmed through a quantitative study that workload among nurses providing care to patients with COVID-19 might disrupt private life and decrease quality of life (27). A decrease in the quality of life of nurses caring for patients with COVID-19 has also been confirmed by Turkish researchers (28). Studies based on qualitative methodology, such as that of Barello et al. (10), offer rich descriptions of the emotional and psychological burdens experienced by nurses. These findings complement our quantitative results by providing context for the reported stress levels and pointing to the personal impact of systemic shortages. Our stuy of nurses experienced stress during the care of patients with COVID-19 (64.7%), and 33.6% of participants experienced stress ‘sometimes’ (33.6%). Nurses with secondary school education and those aged 41–50 years reported exposure to stress significantly more frequently. Psychosocial factors, such as stress and burnout, play a significant role in the working conditions of nurses. Studies, such as those by Kowalczuk et al. (29), have shown how hazardous working conditions can impact nurses’ well-being, exacerbating the challenges they face in their roles. Recent studies, such as the editorial by Jerg-Bretzke et al. (30), discuss updated international findings on the psychological burden of healthcare workers during the pandemic, providing a broader context for your results. As the sources of stress, the nurses most often mentioned risk of infection with SARS-CoV-2 (85.8%), severe state of health of patients (49.6%), shortages of equipment (44.2%), and patients’ death while on duty (44.2%). In relation to stress, the examined nurses most frequently indicated complaints such as difficulty concentrating (70.9%), sleep problems (64.5%), and headaches (56.4%).

Researchers from Portugal (Carvalhais, Querido, Pereira, and Santos) have emphasized that infection with SARS-CoV-2 is an important hazardous factor during the care of patients with COVID-19. In their opinion, the risk of infection with SARS-CoV-2 should be included in OSH practice [personal protective equipment, training, assessment of occupational risk (biological)] (31, 32). The study showed that the largest number of nurses in the study confirmed the occurrence of hazardous factors during the care of patients with COVID-19 (69.8%). Recent multicenter European studies have emphasized the role of immunological protection in reducing infection risk among healthcare workers. For instance, Spiteri et al. (33) demonstrated that higher anti-S IgG antibody levels were correlated with lower rates of breakthrough infections, particularly in the post-Omicron phase. Similarly, Violan et al. (34) highlighted how multimorbidity affected long-term antibody responses and contributed to occupational vulnerability. In the first place, the participants mentioned the occurrence of biological factors (84.3%), followed by physical (73.0%) and chemical factors (63.5%). During the care of patients with COVID-19, the participants also observed the occurrence of arduous factors (69%). As the main arduous factors, the nurses in the study indicated psychological load (95.7%), followed by physical effort (83.6%) and shift work (53.4%).

In the opinion of researchers Ranney, Griffeth, and Jha, at the beginning of the COVID-19 pandemic, American hospitals reported shortages of equipment (respirators) during the care of patients infected with SARS-CoV-2 (35). The present study demonstrated that, according to the majority of nurses (74.1%) providing care to patients with COVID-19, the wards were ‘sometimes’ provided with proper equipment/devices. In the opinions of this group of participants, shortages mainly concerned respirators (88.8%). Apart from this, 15.5% of the participants considered that hospital wards were always provided with proper equipment/devices, while 10.3% of the participants considered that they were not.

The goal of the survey was achieved because it allowed recognition of the conditions of nurses’ work in the care of patients with COVID-19. The results of this study may be applied in practice among management staff to improve the organization of the work of nursing staff providing care to patients infected with a dangerous pathogen. In addition, the results of this study should provide incentives for researchers in other medical professions to conduct in-depth studies in order to optimize work conditions during the treatment of patients infected with SARS-CoV-2 in hospital wards.

### 4.1 Limitations

This study has several limitations. First, although the epidemic threat in Poland officially lasted until June 30, 2023, data collection took place between February 1 and March 3, 2023, a period corresponding to the late phase of the COVID-19 pandemic, commonly described in recent European studies as the “post-Omicron” phase. This period was marked by widespread community transmission, high vaccination coverage (including boosters), and reduced clinical severity of cases. As a result, the epidemiological context during data collection differed significantly from the earlier phases of the pandemic, such as the pre-vaccine or Delta periods. Dedicated COVID-19 wards were no longer operating, and the patients were treated in various departments. Consequently, while this study captures important information about nurses’ working conditions, it does not allow for phase-stratified comparisons. This temporal limitation should be considered when interpreting the findings.

Second, the relatively short data collection period may not have fully captured the changes in working conditions or infection risk throughout the different phases of the pandemic. Third, the study relied on retrospective self-reported data, which introduced the risk of recall bias, particularly concerning perceived stress levels and work-related experiences.

In addition, key variables, such as COVID-19 vaccination status, history of SARS-CoV-2 infection, and the presence of long-COVID symptoms, were not assessed. These unmeasured factors may act as confounders that influence participants’ perceived biological exposure risk and psychological burden. Future studies should include these factors and be conducted on a larger and more diverse sample across multiple institutions.

## 5 Conclusion

Nurses employed in hospital wards during the care of patients with COVID-19 in Poland operated under highly demanding and often insufficient working conditions. Personal protective equipment (PPE) was inconsistently provided, and many nurses reported a lack of adequate training regarding its proper use. Staffing levels were frequently below regulatory standards, with the most acute shortages occurring during night shifts, on non-working days, and holidays.

The study participants reported substantial exposure to stress, primarily caused by the fear of infection with SARS-CoV-2, the critical condition of patients, patient deaths during shifts, and the limited availability of necessary equipment. The findings also highlighted a wide spectrum of occupational hazards—biological, physical, and chemical—as well as arduous factors such as psychological burden, physical strain, and shift work. Moreover, shortages of essential medical equipment, including respirators, cardiac monitors, and inhalators, further intensified the challenges encountered by nurses.

These findings underscore the urgent need for systemic improvements in hospital preparedness and nurse support, including.

- Consistent and adequate provision of PPE.
- Ensuring compliance with staffing standards.
- Full access to critical medical equipment.
- Comprehensive training on protective procedures.
- Psychological support mechanisms for nursing staff.

Addressing these areas is essential not only for improving nurses’ working conditions during health crises but also for safeguarding their physical and mental well-being. Future research should investigate the long-term psychological impact of the COVID-19 pandemic on nurses and inform the development of preparedness strategies for future public health emergencies.

## Data Availability

The raw data supporting the conclusions of this article will be made available by the authors, without undue reservation.
